# A longitudinal study of facial function, quality of life, and depression in Bell’s palsy during pregnancy and puerperium

**DOI:** 10.1038/s41598-024-75552-5

**Published:** 2024-10-22

**Authors:** Lovisa Lansing, Sophia Brismar Wendel, Ellen Wejde Westlund, Elin Marsk

**Affiliations:** 1 Department of Otorhinolaryngology, Karolinska University Hospital, 141 86 Huddinge, Sweden; 2 Department of Clinical Science, Intervention and Technology, Karolinska Institutet, 141 52 Huddinge, Sweden; 3 Department of Clinical Sciences, Danderyd Hospital, Karolinska Institutet, 182 88 Stockholm, Sweden; 4 Department of Women’s Health, Danderyd Hospital, 182 88 Stockholm, Sweden

**Keywords:** Peripheral facial palsy, Facial paralysis, EPDS, FaCE scale, FDI, Sunnybrook, Epidemiology, Peripheral neuropathies, Quality of life, Clinical trials

## Abstract

Bell’s palsy can reduce facial function and quality of life. Pregnancy may also be physically and psychologically challenging. This study investigates depression among pregnant and puerperal women with and without Bell’s palsy and if degree of facial palsy and depression was correlated. Thirty-one women with pregnancy-associated Bell’s and 31 women without Bell’s palsy were prospectively included and followed one year at two University Hospitals, Stockholm. Depression was assessed with Edinburgh Postnatal Depression Scale (EPDS). In women with Bell’s palsy, Facial Disability Index (FDI), Facial Clinimetric Evaluation (FaCE) scale, and Sunnybrook Facial Grading System (SFGS) were collected. The association between Bell’s palsy and EPDS ≥ 11 was assessed by logistic regression, and between EPDS and FDI, FaCE, and SFGS, respectively, by Spearman rank correlation. Median EDPS did not differ between groups (7.0 vs. 6.0, *p* = 0.74, one month, 6.5 vs. 6.0, *p* = 0.87, 12 months). EPDS at one month was correlated to FDI (*p* = 0.002) and FaCE (*p* = 0.004) and at 12 months to FDI (*p* = 0.009) but not to FaCE (*p* = 0.08). No correlation was found between EPDS and SFGS. In summary, no association appeared between pregnancy-associated Bell’s palsy and depression. Patient-reported function correlated well with depression in pregnancy-associated Bell’s palsy, while physician-reported facial function did not.

**Supplementary Information:**

The online version contains supplementary material available at 10.1038/s41598-024-75552-5.

## Introduction

Bell’s palsy is an idiopathic peripheral facial palsy with sudden onset, leading to weakness or total paralysis of the mimic muscles of the face^1^. This can result in facial asymmetry, oral incompetence, problems closing the eye, and excessive tear flow or dryness of the eye with subsequent corneal injury^2^. In addition, Bell’s palsy may lead to taste disorders, phonophobia, postauricular pain^1^, and psychological problems^3^.

In the general population, the estimated annual incidence of Bell’s palsy has been reported to 23–53 per 100,000 person-years^1,4,5^. Among pregnant and puerperal women, higher incidence numbers have been reported, especially in the third trimester of the pregnancy^6–9^. Even if full recovery is seen in up to 70% of patients^1^, 30% have residual symptoms with varying degrees of facial weakness and/or synkinesis (involuntary movements) resulting in functional and aesthetic deficits. Decreased asymmetric facial movements and possible synkinesis impair the ability to express emotions, affecting social and physical functioning, with unfavourable impact on quality of life^3,10^.

Pregnancy can generate physical and emotional distress such as nausea and vomiting, back pain, gastroesophageal reflux, constipation, sleep disorder, and anxiety for possible complications during pregnancy and childbirth. Puerperium (first six weeks after childbirth)^11^ may also be strenuous with recovery after a possible complicated childbirth, breast-feeding adaptation, sleep disorder, postpartum depression, and adjustment to a new family situation, all of which may impact the quality of life^12–15^. Pregnancy and puerperium are periods in life where expectations of happiness are high, both from the woman herself but also based on social norms^16^. The perceived social conventions and external expectations are often expressed in social activities with family and friends^17–19^. Having a facial palsy under these circumstances might be challenging both cosmetically and physically, and expectations on social interactions might not be fulfilled. Also, a negative effect on communication and attachment could be assumed since the mother no longer wants to smile on photos and towards the child, and consequently the mother-child dyad could be at risk. However, no studies have focused on this question so far.

A woman’s mood and quality of life may be negatively affected by both Bell’s palsy^3,10^, pregnancy^12,20^ and puerperium^13,21^, therefore we hypothesized that women with Bell’s palsy during pregnancy and puerperium could experience a greater negative impact on mood and quality of life than women with one of these states alone. To our knowledge, no studies have assessed the association between pregnancy-associated Bell’s palsy and depression.

The primary aim of this study was thus to compare the degree of depression among women with Bell’s palsy during pregnancy and puerperium to that of pregnant and puerperal women without facial palsy. Furthermore, in this paper we compared patient-reported facial and psychosocial function, patient-reported facial palsy-specific quality of life and physician-reported facial function respectively with a patient-reported questionnaire for pregnancy-related depression, among pregnant and puerperal women with Bell’s palsy.

## Methods

This is a prospective cohort study from Stockholm, Sweden. Women with Bell’s palsy in pregnancy and puerperium referred to the Department of Otorhinolaryngology at the Karolinska University Hospital in Stockholm, Sweden, were included in the exposure group from August 2019 until November 2021. This department is the only tertiary referral centre for facial palsy patients in the Stockholm region with approximately two million inhabitants. Referrals were sent from primary care centres, as well as obstetrics and gynaecology departments, as well as emergency departments in the Stockholm region. Inclusion criteria were as follows: being diagnosed with Bell’s palsy, being pregnant or puerperal with a childbirth during the last six weeks and being able to speak and read Swedish or English. The women with Bell’s palsy were treated according to local treatment praxis, including a full otorhinolaryngological examination to exclude other causes of peripheral facial palsy, including Borrelia Burgdorferi infection. After obstetric consultation, they were treated with tapered per oral corticosteroids for 10 days if no contradictory factors prevailed. All patients with Bell’s palsy were, according to the local care program, followed up at 1 month and when remaining symptoms of the facial palsy at 3, 6 and 9 months, respectively. All patients were, regardless of remaining symptoms of their facial palsy, followed up at 12 months for the longitudinal perspective of the study. None of the women received botulinum toxin treatment.

Background information on pregnancy and childbirth, including number of pregnancies and childbirths, in vitro fertilization, previous and current medical conditions, history of depression, heredity for depression, smoking, and occupation, was collected by interview.

The grade of the palsy was assessed at one and 12 months after onset using the Sunnybrook Facial Grading System. This evaluation tool compares the affected side of the face with the normal side and assesses facial symmetry at rest, symmetry of voluntary movement, and potential synkinesis associated with voluntary movements. Three sub scores are obtained and a composite score is tabulated ranging from 0 to 100, where 0 is total paralysis and 100 is normal facial functioning^22^.

The included women were asked to fill out three validated self-graded questionnaires regarding the impact on mood and facial function: the Edinburgh Postnatal Depression Scale (EPDS), the Facial Disability Index (FDI), and the Facial Clinimetric Evaluation (FaCE) scale. The questionnaires were filled out at one and 12 months after the onset of the facial palsy.

EPDS is a validated self-assessment questionnaire regarding state of mind that is used during pregnancy and puerperium to screen for depression and anxiety^23–25^. It is designed to exclude the effect of concomitant factors correlated to pregnancy and puerperium^21^. The questionnaire contains ten questions concerning mental mood during the previous seven days, with scores from 0 (best) to 3 (worst) resulting in total scores from 0 to 30. EPDS has been validated for use in most countries in the world^26^. In Sweden, EPDS is used at maternal health centers during pregnancy^14,21^, and at Children’s Health Centers around 6–8 weeks postpartum, according to the recommendation of The Swedish National Board of Health and Welfare^27^. The suggested optimal cut-off to detect depression is ≥11 or ≥ 13 points, with increasing sensitivity with a lower value^23,24,28^.

FDI is a disease-specific, self-graded instrument for evaluating physical disability and social and emotional well-being in patients with facial nerve disorders^29^. The questionnaire is divided into two parts containing 10 questions each. One part evaluates subjective physical function with scores between − 25 (worst) to 100 (best). The second part grades social functioning with scores ranging from 0 (worst) to 100 (best)^29^.

The FaCE scale is also a patient-reported instrument developed for the assessment of health-related quality of life in patients with facial nerve palsy. The questionnaire contains 15 questions measuring facial impairment referring to physiological and anatomical problems, facial disability referring to functional issues such as problems with eating and communication, as well as social and emotional issues secondary to facial nerve injury^30^. The scale is divided into six domains: facial movement, facial comfort, oral function, eye comfort, lacrimal control, and social function. The total score ranges from 0 (worst) to 100 (best)^30^.

As the comparison group, women in pregnancy and puerperium without Bell’s palsy were prospectively recruited from the Department of Obstetrics and Gynaecology at Danderyd Hospital, Stockholm, Sweden. Danderyd Hospital has 6500 annual births, including women from a heterogenic socioeconomic background and with mixed obstetric risk. Inclusion criteria were ongoing pregnancy or giving birth during the previous six weeks, speaking Swedish or English, and no history of facial palsy. Recruitment was made between June 2021 and November 2021 by one gynaecologist connected to the research team. Patients were invited into the study when visiting the hospital for planned or unplanned reasons related to the pregnancy, childbirth, or postpartum period. Each participant in the comparison group was matched to a participant in the exposure group regarding age (+/- two years) and pregnancy week or postpartum week (+/- two weeks). Participants in the comparison group were asked to fill in the EPDS at the same time points as the exposure group. The same background information about the pregnancy and childbirth was collected by interview and from computerized medical records.

Pregnancy week for the cases was registered at the time of Bell’s palsy diagnosis, while the degree of the palsy and questionnaires regarding facial function and depression were evaluated during the one-month follow-up. When matching the controls, four weeks were consequently added to the registered gestational week so that matched cases and controls filled in the questionnaires at the same gestational week. Therefore, some of the pregnant cases that were in the last weeks of pregnancy were matched to controls that had recently given birth.

This study was approved on March 9, 2016, by the Regional Ethical Review Board in Stockholm (2015/2349-31) with an approved amendment on 23 September 2020 (2020 − 01295). All research was performed in accordance with relevant regulations and informed consent was obtained from all participants. This study is reported according to the STROBE checklist (supplementary material)^31^.

### Statistical analyses

Background characteristics were described by mean value and standard deviation for age, and numbers and proportions for categorical variables. Maternal age was also categorized into < 35/≥35 years, and parity into primiparous for first-time mothers and multiparous for women with previous childbirths. Trimester was categorized into 1st (gestational week 1–12^+6^), 2nd (gestational week 13^+0^-28^+6^) or 3rd (gestational week 29^+0^ or more). Intercurrent disease was defined as any of the following conditions (binary): hypertension/preeclampsia, all types of diabetes mellitus, hypothyroidism, and multiple sclerosis. Background characteristics were compared between women with and without Bell’s palsy using t-test for age (continuous, normally distributed) and Chi^2^ test for categorical variables or Fisher’s exact test for categorical variables with rare events (< 5).

EPDS was close to normally distributed and described by both medians with interquartile range (IQR) in addition to means with standard deviation. EPDS was also categorized into EPDS < 11/≥11 described by numbers and proportions. Test of normality indicated that FDI total, FDI social and FaCE at one month were normally distributed. The rest of the FDI, FaCE, and Sunnybrook Facial Grading System scores were not normally distributed and therefor all scores were described with medians and interquartile range.

**Fig. 1:**
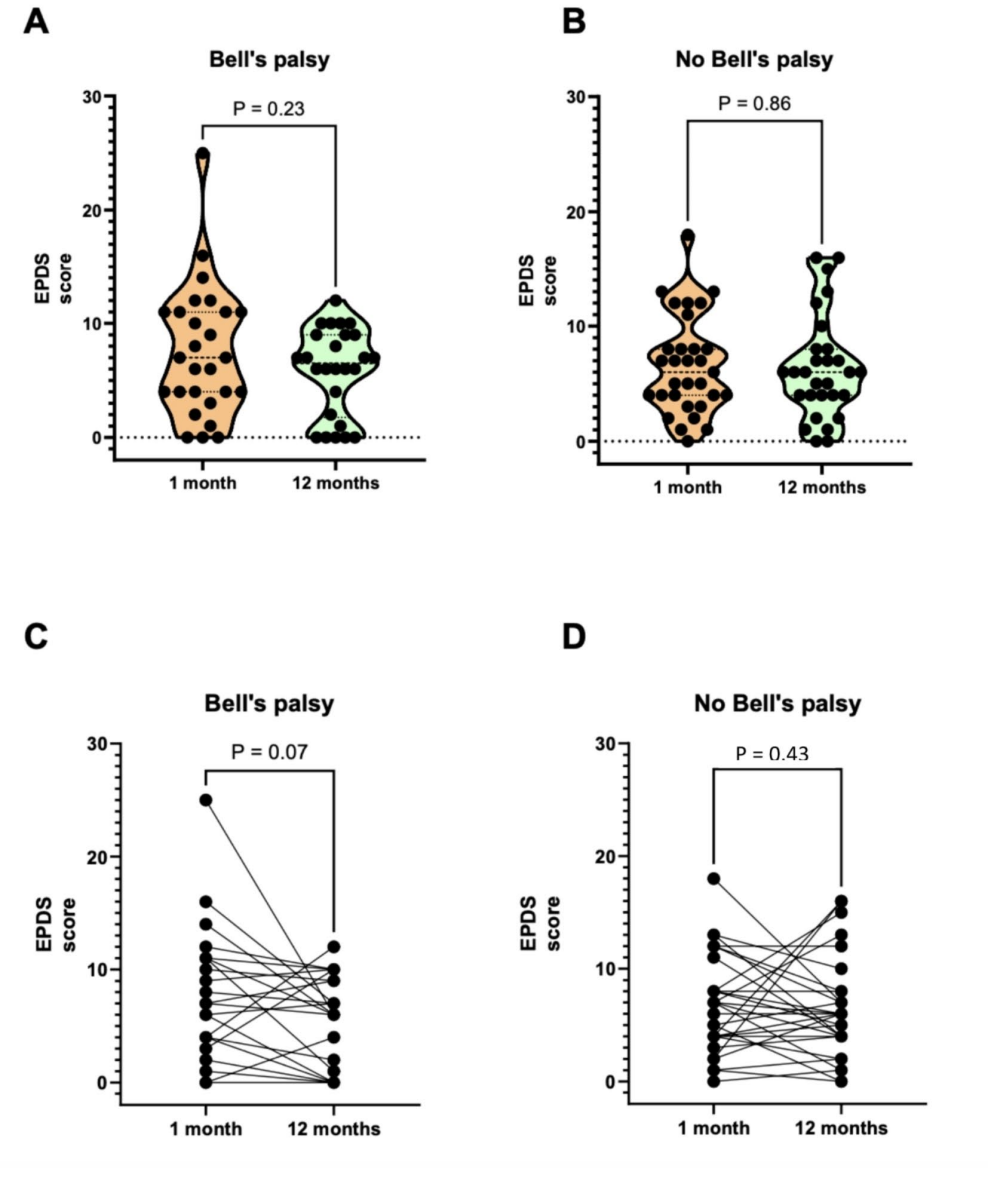
(**a**) Student’s t-test comparing EDPS scores at one and 12 months in pregnant and puerperal women with Bell’s palsy. (**b**) Student’s t-test comparing EDPS scores at one and 12 months in pregnant and puerperal women without Bell’s palsy. (**c**) Wilcoxon signed rank test comparing EPDS scores within-subjects (at one and 12 months), pregnant and puerperal women with Bell’s palsy. (**d**) Wilcoxon signed rank test comparing EPDS scores within-subjects (at one and 12 months), pregnant and puerperal women without Bell’s palsy.

**Fig. 2:**
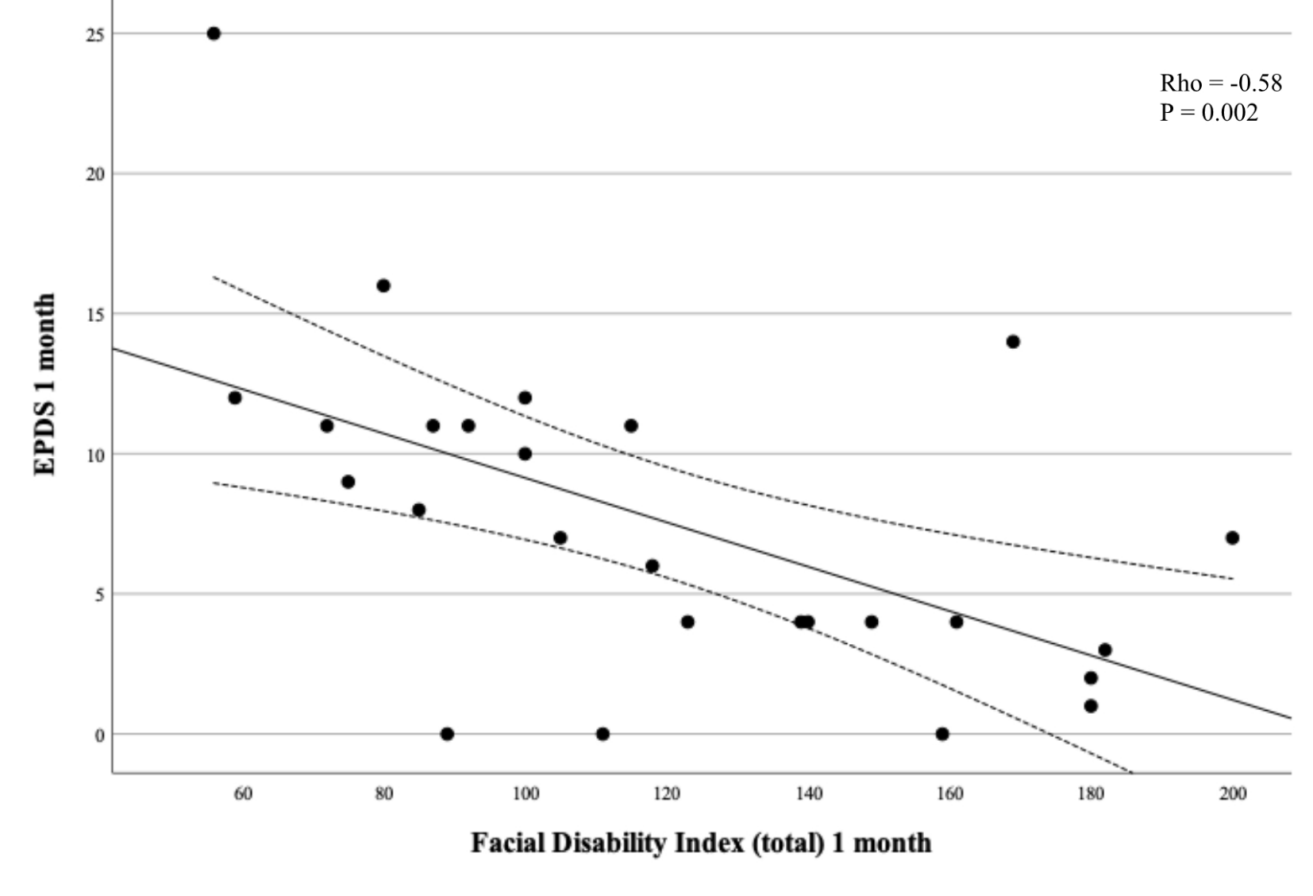
Edinburgh Postnatal Depression Scale (EPDS) as a function of Facial Disability Index (total) at one month.

**Fig. 3:**
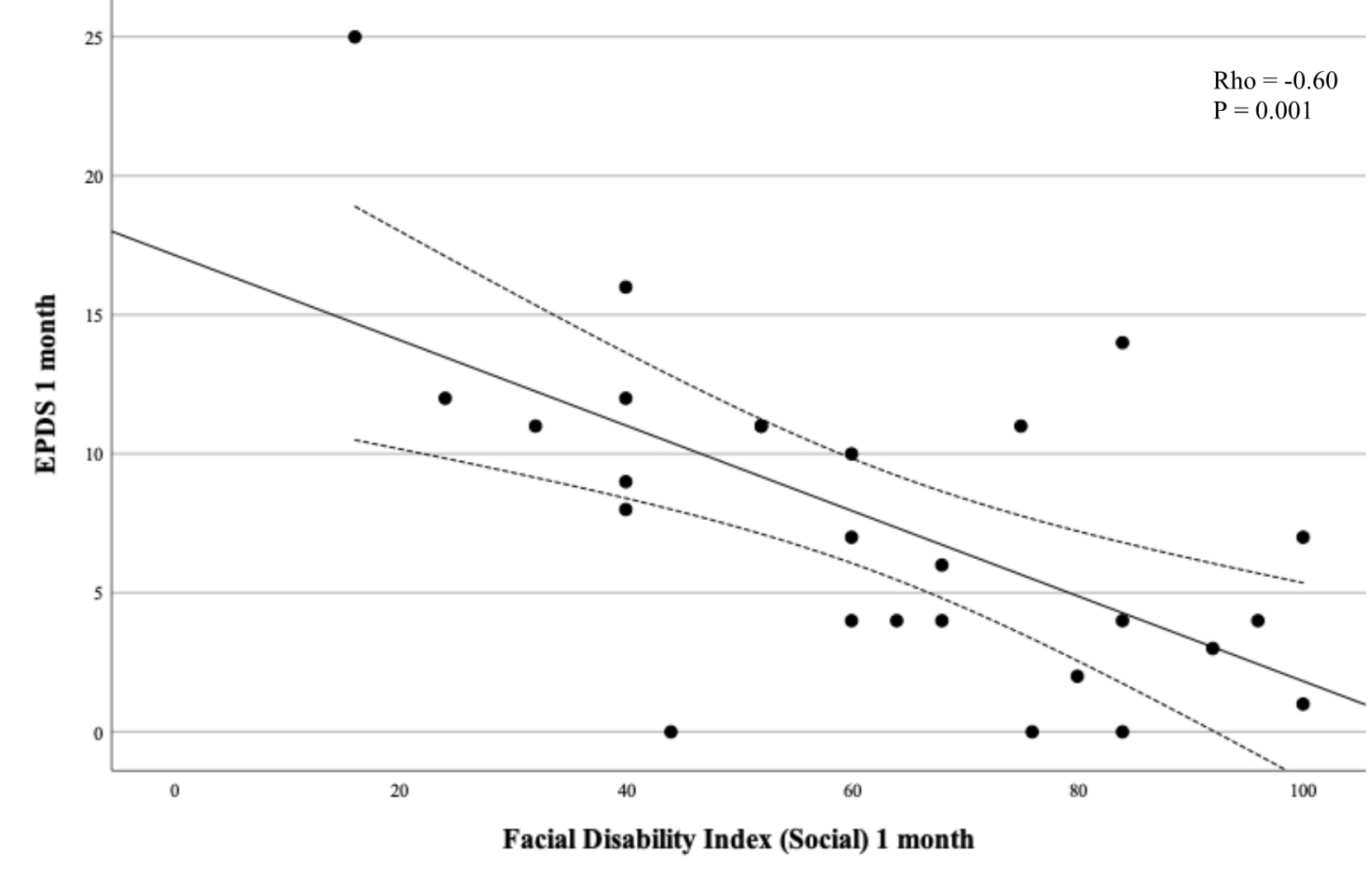
Edinburgh Postnatal Depression Scale (EPDS) as a function of Facial Disability Index (social/well-being subscale) at one month.

**Fig. 4:**
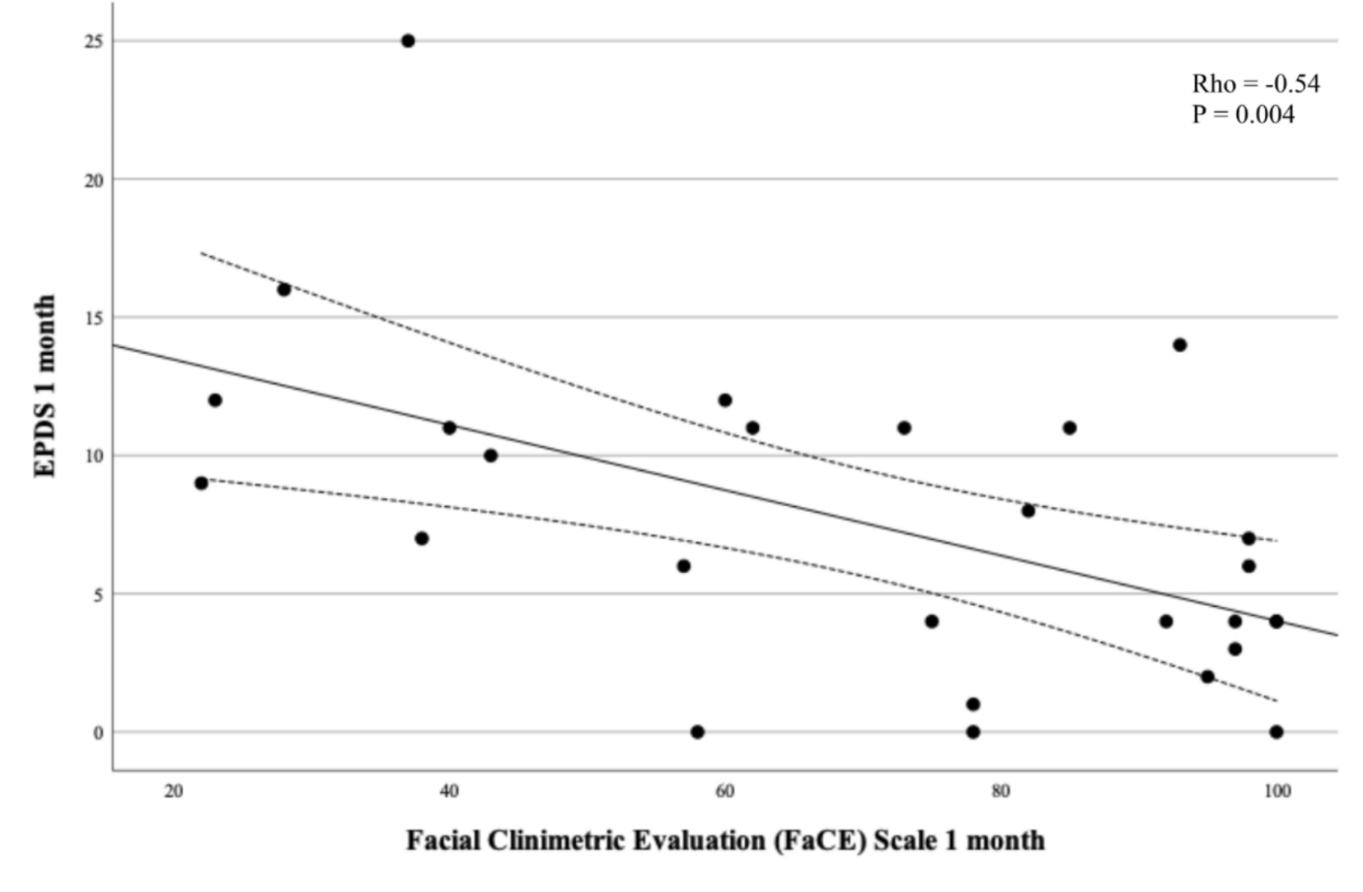
Edinburgh Postnatal Depression Scale (EPDS) as a function of Facial Clinimetric Evaluation (FaCE) Scale at one month.

EPDS medians were compared between women with and without Bell’s palsy using Mann-Whitney test and means with t-test for continuous EPDS. For categorical EPDS < 11/≥11 we used Chi^2^ or Fisher’s exact test for rare events. A Wilcoxon signed rank test was used to compare the within-subjects measures of EPDS at one and 12 months. The association between EPDS < 11/≥11 and Bell’s palsy was analysed using univariable and multivariable logistic regression with complete case analyses. In the multivariable analysis, adjustment was made for previous depression, intercurrent disease as defined above, and in vitro fertilization based on the variation in these variables between the two groups. The results are presented as odds ratios (OR) and adjusted odds ratios (aOR) with 95% confidence intervals (CI). Associations between the EPDS score and FDI, FaCE, and Sunnybrook Facial Grading System respectively, were described by scatter plots and analysed by Spearman rank correlation test at one and 12 months with complete case analyses. No pre-test sample size calculation was made. This study is to be regarded as a pilot study. Analyses were made in IBM SPSS Statistics for Mac, Version 28.0, Armonk, NY: IBM Corp and GraphPad Prism 10.1.2 (GraphPad Software, La Jolla, CA, USA). Figure 1 was made in GraphPad Prism 10.1.2 (GraphPad Software, La Jolla, CA, USA). Figures 2, 3 and 4 were made in Microsoft Excel for Mac (version 16.79.1).

## Results

Initially, 34 pregnant or puerperal women with Bell’s palsy and 34 pregnant or puerperal women without Bell’s palsy were included in the study. Three women with Bell’s palsy withdrew their participation and their matched comparisons were thus excluded. This left 31 women with Bell’s palsy and 31 women without Bell’s palsy participating in the study.

Among cases, two women were lost to 1-year follow-up. Another three of the cases were followed up at 1 year, but never filled in their scores. Among the comparison group, two persons were lost to follow-up (Table 3).

Background characteristics are presented in Table 1. The two groups were similar regarding age, parity, smoking, intercurrent diseases, and pregnant or puerperal status. Among women with Bell’s palsy, 32.3% (10/31) had a previous history of depression, which was non-significantly more than in women without Bell’s palsy (25.8%, 8/31). Women without Bell’s palsy were more often pregnant after IVF (22.6% vs. 3.2%), although the difference was just short of statistical significance (*p* = 0.06). Bell’s palsy occurred during pregnancy in two thirds of the cases, and mostly in the third trimester (around 85%) (Table 1). None of the women reported previous Bell’s palsy, neither did they report herpes infection or borrelia burgdorferi which could cause facial palsy. No other factors that could affect the EPDS score (for example severe pain, certain medications or sociodemographic status) except from earlier or current depression, were reported.

**Table 1:** Background characteristics in pregnant and puerperal women with Bell’s palsy versus pregnant and puerperal women without Bell’s palsy.

	Bell’s palsy *N* = 31 *n* (%)	No Bell’s palsy *N* = 31 *n* (%)	*p*-value
Age (years), mean [SD]	30.5 [4.13]	30.4 [4.06]	0.98
< 35	26 (83.9)	26 (83.9)	1.00
≥ 35	5 (16.1)	5 (16.1)	
Parity			
Primiparous	15 (48.4)	17 (54.8)	0.92
Multiparous	13 (41.9)	14 (45.2)	
Missing	3 (9.7)	0	
Smoking	1 (3.2)	0	0.28
Missing	4 (12.9)	0	
IVF	1 (3.2)	7 (22.6)	0.06
Missing	4 (12.9)	0	
Previous depression	10 (32.3)	8 (25.8)	0.36
Missing	4 (12.9)	0	
Pregnant/Postpartum			
Postpartum	10 (32.3)	15 (48.4)	0.20
Pregnant	21 (67.7)	16 (51.6)	0.20
Trimester			0.96
1st	1 (5.0)	1 (6.3)	
2nd	2 (10.0)	2 (12.5)	
3rd	17 (85.0)	13 (81.3)	
Intercurrent disease	11 (40.7)	14 (45.2)	0.74
Missing	4 (12.9)	0	

Results from the EPDS assessment are presented in Table 2. Among women with Bell’s palsy, 29.0% (9/31) scored ≥ 11 points at one month from onset, compared to 22.6% (7/31) among women without Bell’s palsy. Only one woman with Bell’s palsy scored ≥ 11 points at the 12-months follow-up. Compared to women without Bell’s palsy, there was no statistically significant difference in median or mean EPDS or proportion of women with EPDS ≥ 11 points at one or 12 months. This was also reflected in no increased risk presented as adjusted OR (Table 2). Women with Bell’s palsy seemed to start from a higher EPDS score and decrease more at 12 months than women without Bell’s palsy, although these changes were not statistically significant (Fig. 1a-d).

**Table 2:** Edinburgh Postnatal Depression Scale (EPDS) in pregnant and puerperal women with and without Bell’s palsy, presented as EPDS scores < 11 points and ≥ 11 points at one and 12 months.

	Bell’s palsy *N* = 31 *n* (%)	No Bell’s palsy *N* = 31 *n* (%)	*p*-value	Odds Ratio (95% CI)	Adjusted Odds Ratio (95% CI)*
One month					
Median [IQR]	7.0 [4.0–11.0]	6.0 [4.0–8.0]	0.74		
Mean [SD]	7.5 [5.7]	6.6 [4.3]	0.50		
≥ 11 points	9 (29.0)	7 (22.6)	0.36	1.71 (0.54–5.48)	2.47 (0.63–9.69)
< 11 points	18 (58.1)	24 (77.4)		1.0 (ref.)	1.0 (ref.)
Missing	4 (12.9)	0			
12 months					
Median [IQR]	6.5 [1.8-9.0]	6.0 [4.0–8.0]	0.87		
Mean [SD]	5.9 [3.8]	6.4 [4.5]	0.64		
≥ 11 points	1 (3.2)	5 (16.1)	0.20	0.19 (0.02–1.77)	0.13 (0.01–1.50)
< 11 points	25 (80.6)	24 (77.4)		1.0 (ref.)	1.0 (ref.)
Missing	5 (16.1)	2 (6.5)			

IQR = interquartile range. *Adjusted for previous depression, intercurrent disease, and IVF.

Among women with Bell’s palsy, the median patient-reported facial function with FDI was 50.0 (IQR 40.0–75.0) for physical function and 60.0 (IQR 40.0–84.0) for social function at one month. At 12 months, median FDI was 95.0 (IQR 71.3–100.0) for physical and 86.0 (IQR 60.0–96.0) for social, respectively. The median patient-reported facial function with FaCE was 78.0 (IQR 43.0–97.0) at one month and 94.0 (IQR 75.0-100.0) at 12 months. The median physician-reported facial function by Sunnybrook Facial Grading System score at one month was 87.5 (IQR 46.3–99.0) and among them 32% were severe palsies (Sunnybrook Facial Grading System score < 50). At 12 months after onset, the median Sunnybrook Facial Grading System score was 100.0 (IQR 85.0-100.0). Only one patient (3%) had severe Bell’s palsy after 12 months and her Sunnybrook Facial Grading System score at one month was 18. At one month 25% had reached Sunnybrook Facial Grading System score 100 and 46% had Sunnybrook Facial Grading System score > 90. At 12 months the score was 55% versus 72% respectively.

EPDS at one month was significantly inversely correlated to FDI showing higher depression score at lower facial function score (Table 3; Fig. 2). This correlation was significant for FDI total (rho − 0.58, p 0.002), FDI physical (rho − 0.44, p 0.02), and most pronounced between EPDS and FDI social (rho − 0.60, p 0.001, Table 3; Fig. 3). EDPS at one month was also inversely correlated with patient-reported facial function with FaCE at one month (rho − 0.54, p 0.004, Table 3; Fig. 4). EPDS at one month was not correlated to Sunnybrook Facial Grading System score (Table 3). EPDS at 12 months was significantly inversely correlated with FDI total and FDI social but not with the other scores (Table 3).

**Table 3:** Edinburgh Postnatal Depression Scale (EPDS) as a function of different facial assessment scales (Sunnybrook Facial Grading System, Facial Disability Index, FACE) at one month and 12 months, presented with rho values and 95% confidence intervals (CI).

	Rho value	95% CI	*p*-value
One month			
FDI total^a^	-0.58	-0.80 to -0.24	0.002
FDI physical^a^	-0.44	-0.71 to -0.05	0.02
FDI social^a^	-0.60	-0.81 to -026	0.001
FaCE^a^	-0.54	-0.77 to -0.19	0.004
Sunnybrook Facial Grading System^b^	-0.17	-0.54 to 0.25	0.41
12 months			
FDI total^c^	-0.50	-0.75 to -0.13	0.009
FDI physical^c^	-0.33	-0.64 to 0.08	0.10
FDI social^c^	-0.54	-0.77 to -0.17	0.005
FaCE^c^	-0.32	-0.64 to 0.09	0.11
Sunnybrook Facial Grading System^d^	-0.35	-0.66 to 0.05	0.08

^a^4 missing, ^b^3 missing, ^c^5 missing, ^d^2 missing.

## Discussion

In this study, EPDS scores were similar in pregnant and puerperal women with and without Bell’s palsy. Among pregnant and puerperal women with Bell’s palsy, patient-reported function using FDI and FaCE correlated well with the degree of depression (high EPDS score). This association was seen at one month after the onset of Bell’s palsy, when symptoms usually peak. At 12 months after onset, when symptoms usually have decreased, the association was not as strong with FDI and no longer significant with FaCE. The physician-reported facial function scale Sunnybrook Facial Grading System did not correlate well with the EPDS score in this study.

To our knowledge, this is the first study that investigates self-graded depression in patients with Bell’s palsy in pregnancy and puerperium and compares it to pregnant and puerperal women without Bell’s palsy. Previous studies regarding Bell’s palsy in pregnancy and puerperium have mainly focused on incidence^6–8,32,33^, prognosis^1,7,34^, and associated risk factors^8,35–37^. In our study, we found no clear difference in depression score using EPDS between pregnant and puerperal women with Bell’s palsy and without Bell’s palsy.

Among women with Bell’s palsy, the degree of perceived facial dysfunction and quality of life had a clear correlation to pregnancy-related or postnatal depression. Interestingly, the physician-reported facial function was not a good predictor of depression. This is consistent with the findings of Bylund et al., in which there was only a low to fair correlation between how the physician and the patient perceived the situation using the patient-reported FDI/FaCE and the physician-reported Sunnybrook Facial Grading System score one month after onset of Bell’s palsy^10^. In order to assess how a patient functions and feels, our finding supports the use of patient-reported disability, such as FDI^29,38^ and FaCE^39,40^, rather than physician-reported function like the Sunnybrook Facial Grading System. Moreover, the study by Bylund, although not focusing on pregnant and puerperal women, did compare women and men, and found that women experienced greater psychosocial disability in connection to Bell’s palsy than men^10^. This could be related to different social expectations, which may well be accentuated in pregnancy and with a new-born.

In addition to being the first study to investigate pregnancy-related and postpartum depression in patients with Bell’s palsy in pregnancy and puerperium using validated instruments, other strengths of this study are its prospectively collected data and a matched comparison group. By matching for age and gestational week, we avoided confounding related to time in life and time in pregnancy or puerperium, although other interindividual differences may also be important. Thus, the main limitation in this study is the moderate sample size, which entails power problems. Non-significant findings could become significant with a larger sample size. A post-hoc sample size calculation reveals that 732 women in each group would have been needed to confirm the observed difference in EPDS ≥ 11 at one month. As a pilot study based on data from a single centre, the generalizability of the results is limited. Moreover, despite being the only regional referral centre for Bell’s palsy and that we included women consecutively, there is still a risk that we missed cases. These missed cases were likely mild or transient, which, had they been included, would have decreased differences between cases and controls. On the other hand, controls were recruited in an obstetrics and gynaecology department, meaning that they were also likely to have more health issues than the general pregnant population, matching our cases better. Still again, if we had included milder cases of Bell’s palsy, it might have strengthened the correlation between perceived facial palsy and depression scores. Another caveat is that this study took place during the Covid-19 pandemic which is important to consider when analysing mental health. Most women with Bell’s palsy were included during a period when restrictions regarding social interactions prevailed although Sweden had much milder restrictions compared to the rest of the world, while the comparison group was included when most restrictions were written off. This could lead to depression among cases for other reasons than facial palsy and thus overestimate the overall EPDS among them, but should not affect the association between perceived facial palsy and depression scores.

## Conclusion

Similar depression scores were observed in pregnant and puerperal women with and without Bell’s palsy. Patient-reported facial disability and facial palsy-specific quality of life assessment scales correlated well with the degree of depression among patients with Bell’s palsy in pregnancy and puerperium. Physician-reported facial functioning assessment scales were poor indicators of depression in this pilot study. Thus, based on our data, patient-reported facial disability questions should be considered in order to identify patients needing intervention regarding risk of depression.

## Electronic supplementary material

Below is the link to the electronic supplementary material.

Supplementary Material 1

## Data Availability

The data that support the findings of this study are available on request from the corresponding author.
