# Supplementary material for: A longitudinal study of facial function, quality of life, and depression in Bell’s palsy during pregnancy and puerperium
Source: Sci Rep. 2024 Oct 22;14:24890. doi: 10.1038/s41598-024-75552-5 (PMC11496803; doi:10.1038/s41598-024-75552-5)
Supplement: Supplementary file 1 — Supplementary Material 1 [file 41598_2024_75552_MOESM1_ESM.pdf]

STROBE Statement—checklist of items that should be included in reports of observational studies

	Item No.	Recommendation	Page No.	Relevant text from manuscript
**Title and abstract**	1	(*a*) Indicate the study's design with a commonly used term in the title or the abstract	1-2	Prospective, longitudinal
		(*b*) Provide in the abstract an informative and balanced summary of what was done and what was found	2	See abstract
**Introduction**				
Background/rationale	2	Explain the scientific background and rationale for the investigation being reported	3-4	
Objectives	3	State specific objectives, including any prespecified hypotheses	4	
**Methods**				
Study design	4	Present key elements of study design early in the paper	4	Prospective, cohort
Setting	5	Describe the setting, locations, and relevant dates, including periods of recruitment, exposure, follow-up, and data collection	4-5	
Participants	6	(*a*) *Cohort study*—Give the eligibility criteria, and the sources and methods of selection of participants. Describe methods of follow-up	5	
		(*b*) *Cohort study*—For matched studies, give matching criteria and number of exposed and unexposed	7	
Variables	7	Clearly define all outcomes, exposures, predictors, potential confounders, and effect modifiers. Give diagnostic criteria, if applicable	5-7	
Data sources/ measurement	8*	For each variable of interest, give sources of data and details of methods of assessment (measurement). Describe comparability of assessment methods if there is more than one group	5-7	
Bias	9	Describe any efforts to address potential sources of bias	7	
Study size	10	Explain how the study size was arrived at	9	No pre-test sample size calculation, this is a pilot study

Continued on next page

Quantitative variables	11	Explain how quantitative variables were handled in the analyses. If applicable, describe which groupings were chosen and why	8-9
Statistical methods	12	(*a*) Describe all statistical methods, including those used to control for confounding	8-9
		(*b*) Describe any methods used to examine subgroups and interactions	8-9
		(*c*) Explain how missing data were addressed	8-9
		(*d*) *Cohort study*—If applicable, explain how loss to follow-up was addressed	9
		(*e*) Describe any sensitivity analyses	n/a
**Results**			
Participants	13*	(a) Report numbers of individuals at each stage of study—eg numbers potentially eligible, examined for eligibility, confirmed eligible, included in the study, completing follow-up, and analysed	10
		(b) Give reasons for non-participation at each stage	10
		(c) Consider use of a flow diagram	n/a
Descriptive data	14*	(a) Give characteristics of study participants (eg demographic, clinical, social) and information on exposures and potential confounders	10 and Table 1
		(b) Indicate number of participants with missing data for each variable of interest	Table 1
		(c) *Cohort study*—Summarise follow-up time (eg, average and total amount)	10 and Table 1
Outcome data	15*	*Cohort study*—Report numbers of outcome events or summary measures over time	10-11, Table 2
		Case-control study—Report numbers in each exposure category, or summary measures of exposure	
		Cross-sectional study—Report numbers of outcome events or summary measures	
Main results	16	(*a*) Give unadjusted estimates and, if applicable, confounder-adjusted estimates and their precision (eg, 95% confidence interval). Make clear which confounders were adjusted for and why they were included	Table 2
		(*b*) Report category boundaries when continuous variables were categorized	10
		(*c*) If relevant, consider translating estimates of relative risk into absolute risk for a meaningful time period	n/a

Continued on next page

Other analyses	17	Report other analyses done—eg analyses of subgroups and interactions, and sensitivity analyses	11-12, Table 3	
**Discussion**				
Key results	18	Summarise key results with reference to study objectives	12	
Limitations	19	Discuss limitations of the study, taking into account sources of potential bias or imprecision. Discuss both direction and magnitude of any potential bias	13	
Interpretation	20	Give a cautious overall interpretation of results considering objectives, limitations, multiplicity of analyses, results from similar studies, and other relevant evidence	14	
Generalisability	21	Discuss the generalisability (external validity) of the study results	14	
**Other information**				
Funding	22	Give the source of funding and the role of the funders for the present study and, if applicable, for the original study on which the present article is based	n/a	No funding was received for this study

*Give information separately for cases and controls in case-control studies and, if applicable, for exposed and unexposed groups in cohort and cross-sectional studies.

Note: An Explanation and Elaboration article discusses each checklist item and gives methodological background and published examples of transparent reporting. The STROBE checklist is best used in conjunction with this article (freely available on the Web sites of PLoS Medicine at http://www.plosmedicine.org/, Annals of Internal Medicine at http://www.annals.org/, and Epidemiology at http://www.epidem.com/). Information on the STROBE Initiative is available at www.strobe-statement.org.
